# Differential transcriptome profiling of chilling stress response between shoots and rhizomes of *Oryza longistaminata* using RNA sequencing

**DOI:** 10.1371/journal.pone.0188625

**Published:** 2017-11-30

**Authors:** Ting Zhang, Liyu Huang, Yinxiao Wang, Wensheng Wang, Xiuqin Zhao, Shilai Zhang, Jing Zhang, Fengyi Hu, Binying Fu, Zhikang Li

**Affiliations:** 1 Institute of Crop Sciences/National Key Facility for Crop Gene Resources and Genetic Improvement, Chinese Academy of Agricultural Sciences, Beijing, China; 2 School of Agriculture, Yunnan University, Yunnan, China; 3 Research Center for Perennial Rice Engineering and Technology, Yunnan University, Yunnan, China; 4 Shenzhen Institute for Innovative Breeding, Chinese Academy of Agricultural Sciences, Shenzhen, China; Wuhan University, CHINA

## Abstract

Rice (*Oryza sativa*) is very sensitive to chilling stress at seedling and reproductive stages, whereas wild rice, *O*. *longistaminata*, tolerates non-freezing cold temperatures and has overwintering ability. Elucidating the molecular mechanisms of chilling tolerance (CT) in *O*. *longistaminata* should thus provide a basis for rice CT improvement through molecular breeding. In this study, high-throughput RNA sequencing was performed to profile global transcriptome alterations and crucial genes involved in response to long-term low temperature in *O*. *longistaminata* shoots and rhizomes subjected to 7 days of chilling stress. A total of 605 and 403 genes were respectively identified as up- and down-regulated in *O*. *longistaminata* under 7 days of chilling stress, with 354 and 371 differentially expressed genes (DEGs) found exclusively in shoots and rhizomes, respectively. GO enrichment and KEGG pathway analyses revealed that multiple transcriptional regulatory pathways were enriched in commonly induced genes in both tissues; in contrast, only the photosynthesis pathway was prevalent in genes uniquely induced in shoots, whereas several key metabolic pathways and the programmed cell death process were enriched in genes induced only in rhizomes. Further analysis of these tissue-specific DEGs showed that the CBF/DREB1 regulon and other transcription factors (TFs), including AP2/EREBPs, MYBs, and WRKYs, were synergistically involved in transcriptional regulation of chilling stress response in shoots. Different sets of TFs, such as *OsERF922*, *OsNAC9*, *OsWRKY25*, and *WRKY74*, and eight genes encoding antioxidant enzymes were exclusively activated in rhizomes under long-term low-temperature treatment. Furthermore, several *cis*-regulatory elements, including the ICE1-binding site, the GATA element for phytochrome regulation, and the W-box for WRKY binding, were highly abundant in both tissues, confirming the involvement of multiple regulatory genes and complex networks in the transcriptional regulation of CT in *O*. *longistaminata*. Finally, most chilling-induced genes with alternative splicing exclusive to shoots were associated with photosynthesis and regulation of gene expression, while those enriched in rhizomes were primarily related to stress signal transduction; this indicates that tissue-specific transcriptional and post-transcriptional regulation mechanisms synergistically contribute to *O*. *longistaminata* long-term CT. Our findings provide an overview of the complex regulatory networks of CT in *O*. *longistaminata*.

## Introduction

Rice (*Oryza sativa*), one of the most important cereal crops, provides food for more than half of the world’s population. In recent years, rice production has been seriously affected by environmental stresses, including drought, salt, extreme temperatures, and biotic stressors. In terms of stress tolerance, genetic variability in cultivated rice germplasm is very limited; therefore, broadening the useful gene pool for rice is urgently needed [1].

Wild rice relatives are very important genetic resources for the improvement of rice resistance to biotic and abiotic stresses. Among the 20 known wild rice species, *O*. *longistaminata* has been identified as a potential gene donor for a number of valuable agronomic traits, such as disease resistance [2], high biomass production [3], and chilling tolerance (CT) [4]. Elucidating the genetic and molecular mechanisms of these traits could facilitate the use of these genes for rice improvement. Importantly, *O*. *longistaminata* is a rhizomatous, perennial rice with an AA genome like *O*. *sativa*. Unlike cultivated annual rice varieties, *O*. *longistaminata* is able to overwinter in southern China, where the average winter temperature is approximately 5 to 10°C [5]. The same is true for Dongxiang wild rice (*O*. *rufipogon*), which has been reported to have strong overwintering ability in southern and southwestern China [6,7].

Low temperature is a limiting factor for crop productivity in temperate areas. Rice at seedling and reproductive stages is sensitive to chilling stress triggered by exposure to temperatures between 0 and 15°C [8]. The discovery of useful genes for CT may thus facilitate the improvement of rice crop yield under chilling stress conditions. CT in rice is a quantitative trait, with only a few genes and many quantitative trait loci for CT currently identified [9,10,11,12]. Functional genomics analysis has revealed a number of genes involved in plant CT. The DREB1 (DREB1A, DREB1B, and DREB1C)/CBF-mediated transcription network plays a central role in low temperature tolerance during cold acclimation in Arabidopsis [13]. Even though rice lacks the mechanisms for cold acclimation present in Arabidopsis, components of this CBF cold-response pathway have been identified in the former species. In particular, *OsDREB1* genes have been found to be highly induced in rice by chilling stress, and over-expression of *OsDREB1B* in Arabidopsis can evidently enhance CT [14,15]. In addition, the MYBS3-dependent regulatory pathway has been found to have a complementary functional role during persistent cold stress in rice [16]. Comparative transcriptome profiling has revealed that many genes are differentially expressed in rice under chilling stress in a genotype- and tissue-specific manner [17,18,19]. Although numerous genes with diverse functions related to chilling stress response have been identified, the molecular mechanisms of CT in rice need to be further elucidated. In addition, rice plants used for genome-wide gene expression profiling, in most cases, have been subjected to 4°C temperatures in a growth chamber for only 3 to 72 h [17,18,19]. Very few studies of CT in rice have been based on long-term (more than 3 days) chilling treatment.

In this study, RNA sequencing was used to profile global transcriptome alterations in shoots and rhizomes of *O*. *longistaminata* under long-term (7-day) chilling stress. Different gene sets in diverse functional categories were identified as differentially regulated by chilling in a shoot- and rhizome-specific manner. According to our results, multiple regulatory genes and complex genetic networks are involved in the transcriptional regulation of CT in *O*. *longistaminata* in a tissue-specific manner. Our findings provide a foundation for the elucidation of the molecular mechanisms of CT in *O*. *longistaminata* under long-term low-temperature stress.

## Materials and methods

### Plant material and chilling stress treatment

The material used in this study was an unnamed wild rice accession of *O*. *longistaminata* originally collected from Niger. For the chilling stress treatment, plants at the active tillering stage (60 days after germination) growing in soil tubs were placed in a growth chamber (Beijing ZNYT, Bejing, China) maintained at 4°C (±1°C) under a 12-h light/12-h dark photoperiod. Control plants were grown under the same conditions except at 29°C. After 7 days of chilling treatment, plant tissue samples were collected from both chilled and control *O*. *longistaminata* plants (six independent plants of each). Sampled aerial-shoot tissues included 1–2 cm stem tips (apical meristems), the topmost internodes, and the youngest leaves of each sampled plant, while rhizome samples included rhizome tips (1–2 cm) and the internodes. All collected samples were snap-frozen in liquid nitrogen and stored at −80°C.

### Physiological tests of shoots and rhizomes under chilling stress and control conditions

Total soluble sugar contents were estimated using anthrone reagent [20]. Each 100 mg sample was chopped into pieces and immersed in 20 ml of distilled water in a test tube. After heating the tube in a boiling water bath for 20 min and then cooling to room temperature, distilled water was added to a final volume of 30 ml. A 1 ml aliquot of the extract was transferred to a new tube; to this solution, 5 ml of anthrone reagent was added. The mixture was heated in a boiling water bath for 10 min followed by cooling. The optical density was recorded at 620 nm. Free proline and malondialdehyde (MDA) contents were measured as described in a previous study [21]. Antioxidant enzyme activities of catalase (CAT), peroxidase (POD) and superoxide dismutase (SOD) were determined following the protocol of Bonnecarrère et al. [22]. Ascorbic acid (AsA) and reduced glutathione (GSH) contents were measured using previously reported assay methods [23].

### RNA sequencing and data analysis

Total RNA was isolated using Trizol reagent (Invitrogen, USA) from three biological replicates of sampled aerial-shoot or rhizome tissues collected from six plants. The isolated RNA was then purified and concentrated using an RNeasy MinElute cleanup kit (Qiagen), with RNA quality and concentration determined using a bioanalyzer (Agilent). Equal quantities of total RNA from the three biological replicates of each tissue sample were then pooled for RNA sequencing.

Transcriptome sequencing was performed by CapitalBio (Beijing, China) on an Illumina GA_*IIx*_ next-generation sequencing platform. For mRNA library construction and deep sequencing, RNA samples were prepared using a TruSeq RNA sample preparation kit according to the manufacturer’s protocol. The libraries were qualified on an Agilent 2100 bioanalyzer and quantified on a Qubit fluorometer and by quantitative real-time PCR (qRT-PCR). Cluster formation and sequencing were performed following the manufacturer’s standard cBot and sequencing protocols. Analysis of the RNA libraries by multiplexing sequencing consisted of 101 single-read cycles, followed by 7 index-identification cycles and 101 single-read cycles. Primary data analysis and base calling were performed by the Illumina system software. Low-quality reads were removed using a custom Perl script. The remaining high-quality reads were mapped to the rice reference genome of the Rice Gene Annotation Project at Michigan State University (http://rice.plantbiology.msu.edu/) [24] using TopHat [25] and then assembled into unique transcript sequences using Cufflinks with parameters -g -b -u -o [26]. Cuffcompare was used to compare the assembled transcripts of each library to the reference annotation and to construct a non-redundant transcript data set among libraries. Cuffdiff was then used to detect significant changes in gene expression levels [27].

### Identification of differentially expressed genes (DEGs) and Alternative Splicing (AS) events

Alterations in the expressions of genes between chilling stress and normal growth conditions were quantified and normalized to the number of reads per kilobase of transcripts per million mapped reads (RPKM) [28]. A gene was considered to be differentially expressed if its expression level under chilling stress was significantly higher or lower than that recorded under control conditions according to Fisher’s test based on a |log_2_ RPKM ratio| ≥ 2 and a false discovery rate (FDR) < 0.001.

AS events were identified as described in previous studies [29], with junction sites determined using TopHat software with default parameters [25]. The junction sites detected in a gene were used to determine the different types of possible AS events for that target gene.

### Functional Annotation and *Cis*-element analysis of DEGs

Functional enrichment analysis was carried out using Gene Ontology (GO) annotations for *O*. *sativa* in the agriGO database (http://bioinfo.cau.edu.cn/agriGO/) [30]. A hypergeometric test was used to assess statistical significance. To determine whether a gene was overrepresented, the FDR-adjusted significance level cutoff was set at 0.05. A biological pathway analysis for the tissue-enriched genes was conducted using KOBAS 2.0 (http://kobas.cbi.pku.edu.cn/home.do). A hypergeometric test and the 1995 Benjamini–Hochberg FDR correction method were used with cutoff levels of 0.05. *Cis*-elements in both strands of 1-kb upstream promoter sequences of DEGs were identified by comparison with *O*. *sativa* genes in the PLACE *cis*-element database (http://www.dna.affrc.go.jp/PLACE/) [31] using a Perl script (‘regulatory’) provided by CapitalBio. To identify overrepresentation of putative *cis*-regulatory elements between two groups of genes, two-sample tests of proportion were performed with a significance threshold of 0.05.

### qRT-PCR confirmation of DEGs

qRT-PCR using an ABI Prism 7900 Sequence Detection System (Applied Biosystems) was performed to confirm the expression of 25 DEGs randomly selected from each tissue-specific or tissue-enriched gene group identified from RNA sequencing. Diluted cDNA was amplified using gene specific primers (S1 Table) and SYBR Green Master Mix (Applied Biosystems). The expression levels of the transcripts were normalized against endogenous *Actin* transcripts. Each set of experiments was performed three times, and the delta-delta Ct (ddCt) relative quantification strategy was used to evaluate quantitative variation.

## Results and discussion

### Physiological changes in shoots and rhizomes of *o*. *longistaminata* under 7 days of chilling stress

**Fig 1:**
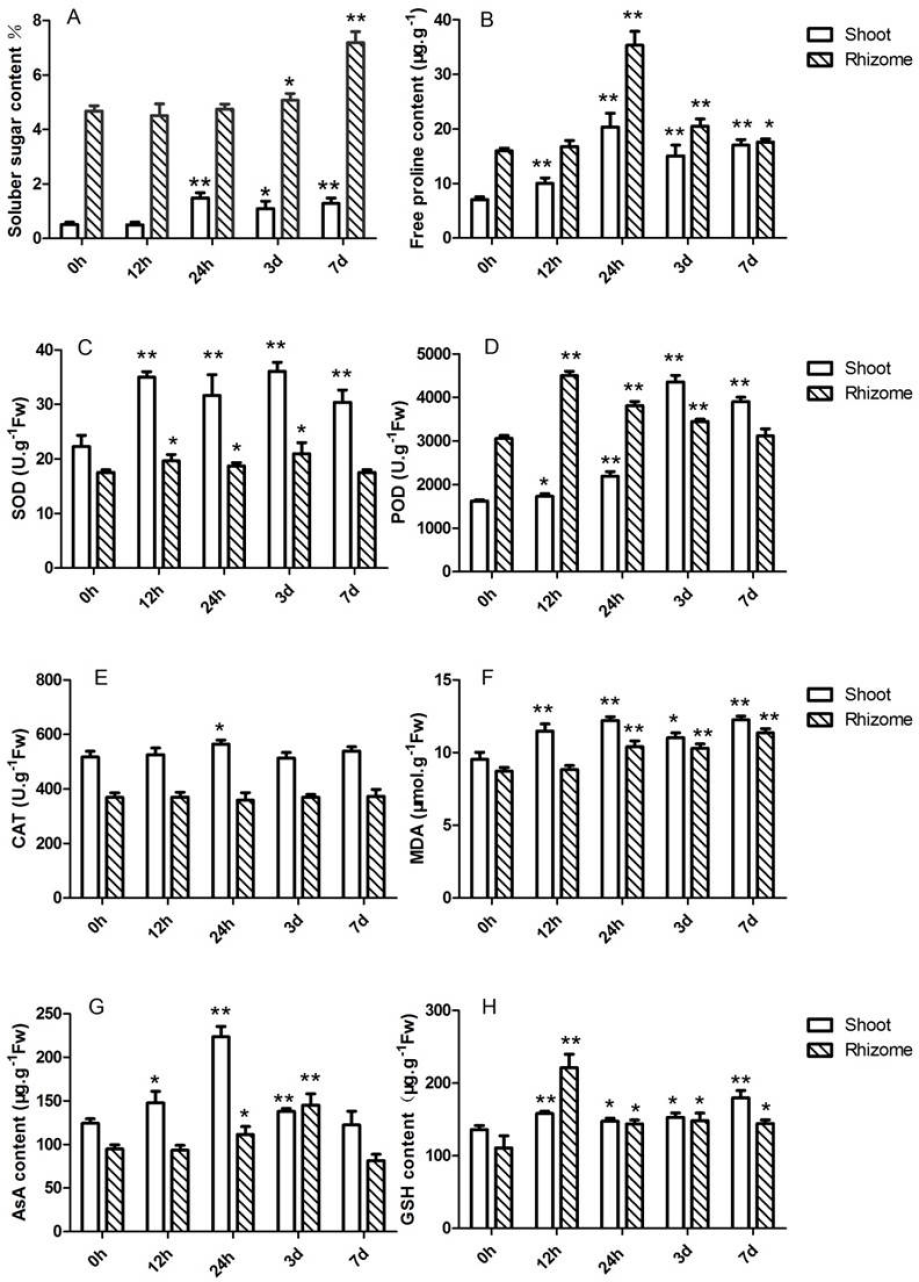
Physiological indexes of shoots and rhizomes of *Oryza longistaminata* after 0 h, 12 h, 24 h, 3 days and 7 days of chilling stress. (A) Soluble sugar content. (B) Free proline content. (C) SOD activity. (D) POD activity. (E) CAT activity. (F) MDA content. (G) AsA content. (H) GSH content. *, *p* ≤ 0.05; **, *p* ≤ 0.01.

*Oryza longistaminata* is a perennial wild rice with overwintering ability in southwestern China [5]. No visible phenotypic differences are evident between *O*. *longistaminata* plants under chilling stress and control conditions. To detect alterations in physiological traits of shoots and rhizomes due to chilling stress, several indices of low temperature-induced effects were therefore measured. As shown in Fig 1A and 1B, shoots and rhizomes, compared with the respective controls, exhibited significantly higher soluble sugar contents after 1-day and 3-day chilling stress, while free proline contents were markedly higher after 12 and 24 h of chilling stress and remained at these levels through the duration of the 7-day treatment. These results demonstrate that *O*. *longistaminata* plants accumulate soluble sugars and free proline in both types of tissues for protection against low temperature stress, with shoots responding more rapidly than rhizomes. Soluble sugars play an important role in the protection of plant cells against damage caused by cold stress by serving as osmoprotectants and nutrients [32,33]. For example, soluble sugar levels have been found to be positively correlated with overwintering in sorghum [34]. In addition, proline accumulation is closely related to abiotic stress tolerance [35], and increased proline content has been observed to be accompanied by a rise in the concentration of soluble sugars [36]. Taken together, these results suggest that increased soluble sugars and free proline in shoots and rhizomes can stabilize biological components and enhance chilling stress tolerance in *O*. *longistaminata*, while the delayed chilling response of these osmoprotectants seen in rhizomes may reflect the fact that this plant organ always grows underground.

Antioxidant enzyme activities and antioxidant concentrations in shoots and rhizomes under chilling stress and control conditions were also comparatively analyzed. Both shoots and rhizomes exhibited a significant increase in SOD and POD activities after 12-h chilling stress compared with the controls, with higher SOD activity detected in shoots than in rhizomes (Fig 1C and 1D). In contrast, CAT activity was stable under both chilling and control conditions in rhizomes and was only increased in shoots at the 24-h chilling stress time point compared with the control. MDA levels were obviously elevated in shoots and rhizomes after 12-h and 24-h chilling treatment compared with the controls (Fig 1E and 1F). We also found that AsA and GSH contents were markedly higher in both tissues during chilling stress treatment (Fig 1G and 1H). All these results indicate that these antioxidants are involved in CT in *O*. *longistaminata*. Previous studies have revealed that plants have highly efficient enzymatic and non-enzymatic antioxidant defense systems to protect plant cells from oxidative damage caused by abiotic stresses [37]. In the present study, the different patterns of increased accumulation of antioxidants in shoots and rhizomes during chilling stress suggest that aboveground shoots and underground rhizomes have slightly different physiological reactions to low temperature stress.

### Transcriptome profiling of shoots and rhizomes of *o*. *longistaminata* under chilling stress and control conditions

To identify molecular responses to chilling stress, gene transcript levels in *O*. *longistaminata* were measured on an Illumina sequencing platform, with shoot and rhizome samples collected for the transcriptome sequencing analysis from plants subjected to 7-day chilling stress and control growth conditions. A total of 32.1, 29.9, 33.2, and 30.0 million 100-bp paired-end reads were obtained from transcriptome libraries of shoots and rhizomes under stress and control conditions, respectively (Table 1); this corresponded to a total length of over 25 gigabases (Gb), equivalent to approximately 58-fold coverage of the *O*. *longistaminata* genome. All the short reads were aligned to the *O*. *sativa* Nipponbare reference genome. As a result, approximately 80% to 91% of total reads from the four transcriptome libraries were mapped to the rice genome, with more than 62% of the mapped reads localized to exon regions (Table 1). Approximately 10% of total reads could not be mapped, an outcome that can primarily be attributed to sequencing gaps and differences between *O*. *sativa* and *O*. *longistaminata* genome sequences.

**Table 1:** Summary of Illumina transcriptome reads of *Oryza longistaminata* mapped to the genome and genes of *O*. *sativa*.

Read mapping	Reads in shoots under control (%)	Reads in shoots under chilling stress (%)	Reads in rhizomes under control (%)	Reads in rhizomes under chilling stress (%)
Total reads	29,859,837	32,104,690	29,979,833	33,208,203
Total base pairs	6,031,687,074	6,485,147,380	6,055,926,266	6,708,057,006
Total mapped reads	48,156,208 (80.6)	58,627,070 (91.3)	48,814,533 (81.4)	55,600,554 (83.7)
Exon	30,794,764 (63.9)	38,258,895 (65.3)	30,533,256 (62.5)	36591944 (65.8)
Intron	867,710 (1.8)	1,091,636 (1.9)	733,076 (1.5)	1414348 (2.5)
InterGenic	1,194,750 (2.5)	9,732,935 (16.6)	1,244,489 (2.5)	4290434 (7.7)
Spliced	15,298,984 (31.8)	9,543,604 (16.3)	16,303,712 (33.4)	13303828 (23.9)

Out of a total of 37,544 genes annotated in the rice genome [38], we identified 30,858 (82.2%) that were expressed in shoots and rhizomes. Among these expressed genes, 28,609/28,454 and 28,081/27,826 were expressed in shoots/rhizomes under chilling stress and control conditions, respectively. These results demonstrate that a majority of genes in the rice genome are active in both shoots and rhizomes.

### DEGs in shoots and rhizomes under chilling stress

**Fig 2:**
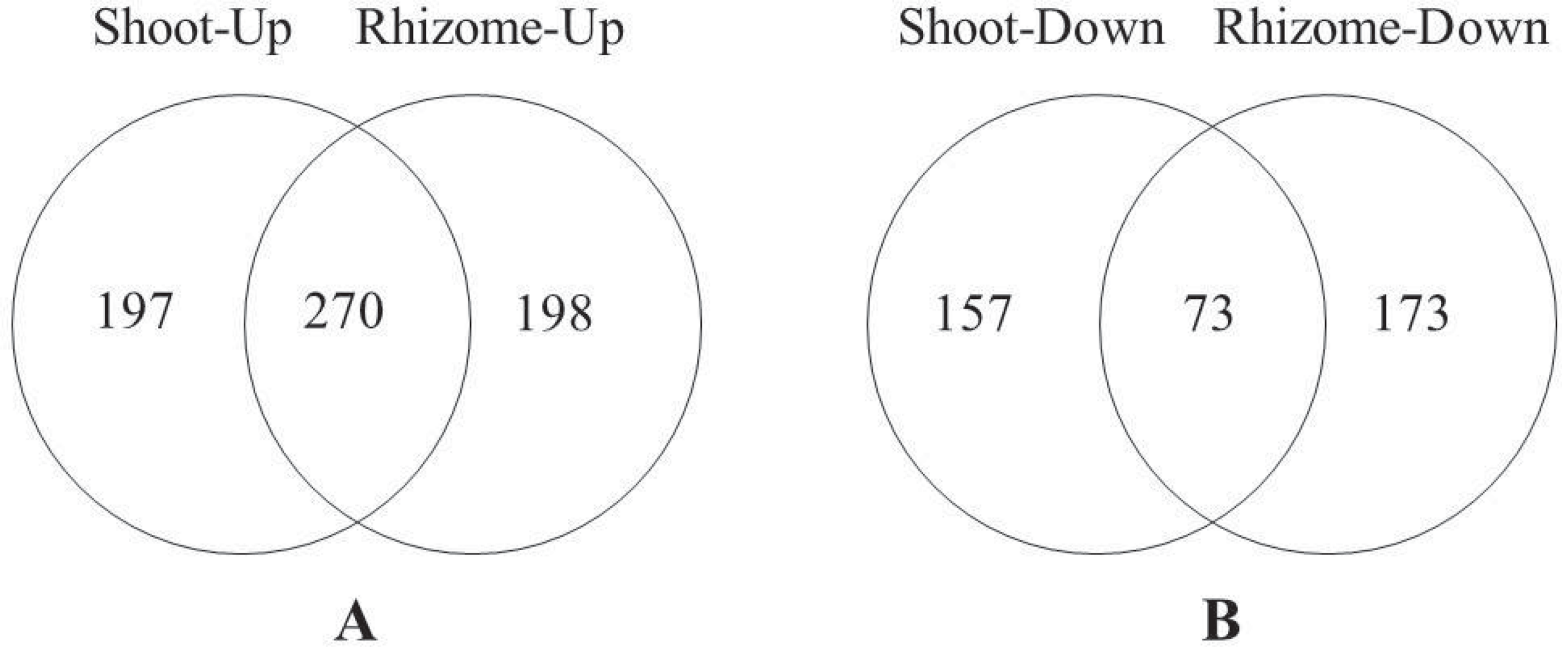
Venn diagram of up- and down-regulated genes in shoots and rhizomes under chilling stress conditions. (A) Up-regulated genes in shoots and rhizomes. (B) Down-regulated genes in shoots and rhizomes.

To explore chilling stress response at the transcriptome level, we screened genes in both shoots and rhizomes for differential expression between chilling stress and control conditions using the criteria of |log_2_ RPKM ratio| ≥ 2 and FDR < 0.001. A total of 697 and 714 genes were respectively identified as differentially expressed in shoots and rhizomes under chilling stress. As shown in Fig 2, 270 and 73 genes were commonly up- and down-regulated, respectively, under chilling stress in both shoots and rhizomes, whereas 197/157 and 198/173 genes were found to be exclusively up-/down-regulated by chilling in shoots and rhizomes, respectively. These results indicate that the transcriptome of *O*. *longistaminata* is evidently reshaped in a tissue-specific manner under long-term chilling stress. To confirm their transcript levels, a set of 25 DEGs were selected and analyzed by qRT-PCR. The gene expression levels uncovered in this qRT-PCR analysis (S1 Fig) were highly similar to those obtained from the transcriptome sequencing data.

**Fig 3:**
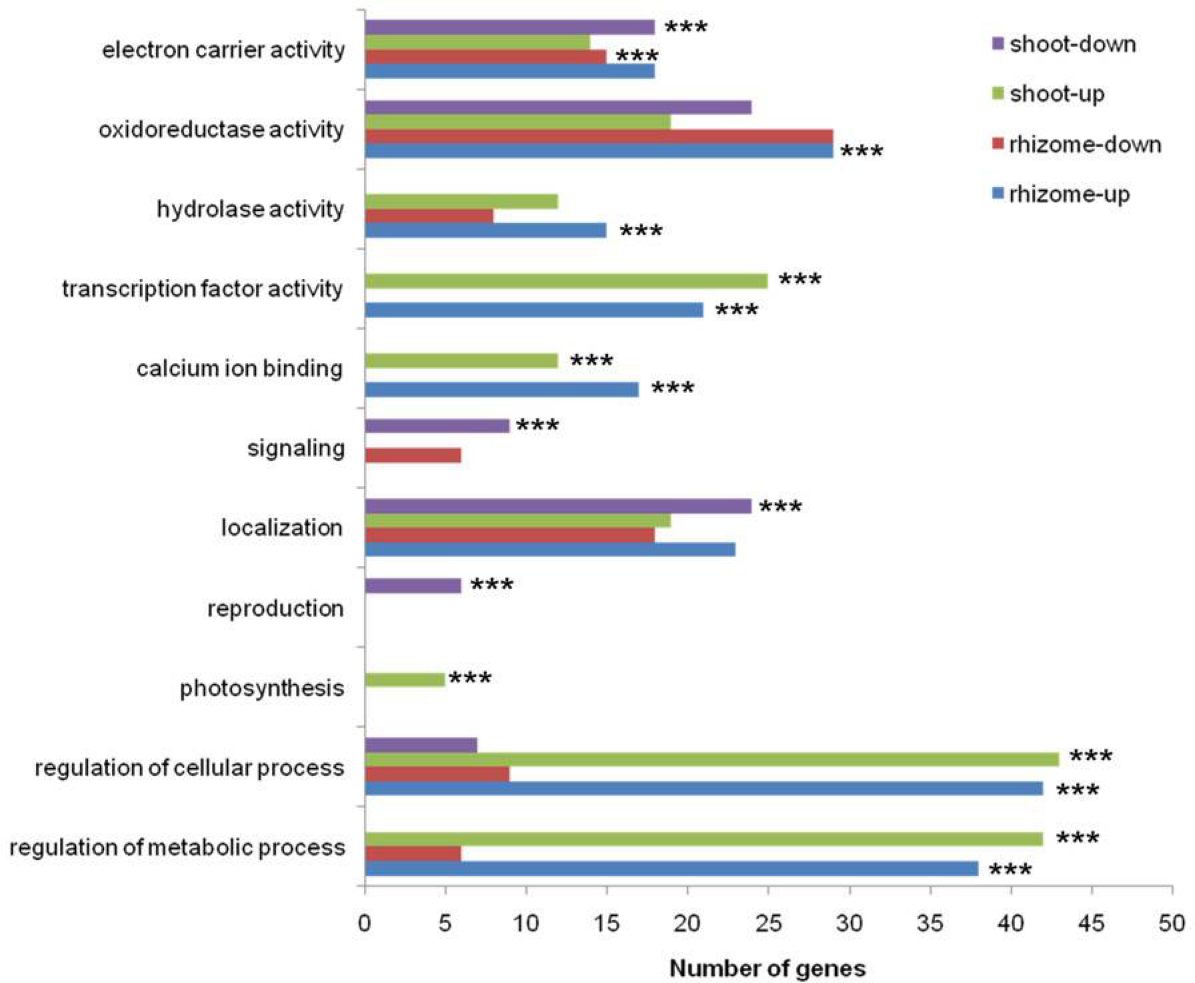
GO slims of functional categories of genes differentially expressed in chilling-treated shoots and rhizomes. The genes were found to be up- or down-regulated in shoots and rhizomes. GO slim categories denoted by *** were significantly overrepresented (*p* ≤ 0.05, hypergeometric distribution).

GO analysis of the above-mentioned DEGs revealed that a functionally diverse set of genes were markedly up- or down-regulated in shoots and rhizomes under 7-day chilling stress conditions (Fig 3). Genes associated with the functional categories of regulation of cellular process, regulation of metabolic process, calcium ion binding, and transcription factor activity were highly enriched among up-regulated genes in both shoots and rhizomes; in contrast, the only commonly enriched down-regulated genes in both tissues were those related to electron carrier activity. Enriched genes related to photosynthesis were specifically detected in the up-regulated set in shoots, while those with hydrolase activity and oxidoreductase activity were uniquely prevalent in the up-regulated set in rhizomes (Fig 3). Taken together, these results indicate that functionally diverse genes are regulated in shoots and rhizomes of *O*. *longistaminata* in response to long-term chilling stress.

### Kyoto encyclopedia of genes and genomes (KEGG) pathway analysis of degs in shoots and rhizomes of *o*. *longistaminata* under chilling stress

To investigate the biological function of chilling-induced genes in shoots and rhizomes of *O*. *longistaminata*, KEGG pathway analysis was performed on genes up-regulated in those organs. The analysis uncovered three pathways, namely, plant–pathogen interaction, plant hormone signaling transduction, and spliceosome, that were evidently enriched in genes commonly induced in both shoots and rhizomes under 7-day chilling stress (Table 2). These results thus functionally implicate genes involved in biotic stress response, phytohormone signaling, and AS in the chilling stress tolerance of *O*. *longistaminata*. Only the photosynthesis pathway was enriched in genes specifically up-regulated in shoots under chilling stress; this implies that photosynthesis was enhanced to withstand the low temperature environment, an observation in agreement with previous reports that overwintering crops can optimize photosynthesis under low temperatures to provide energy necessary for seedling growth and establishment [39,40]. Eight KEGG biological pathways were found to be highly enriched only in genes solely up-regulated in rhizomes, namely, metabolism, tyrosine metabolism, alpha-linolenic acid metabolism, apoptosis signaling, endocytosis, cysteine and methionine metabolism, and plant hormone signal transduction pathways, indicating that these key metabolic pathways and the programmed cell death process were involved in chilling stress response in rhizomes. According to all these results, diverse molecular pathways in shoots and rhizomes are involved in response to long-term chilling stress in *O*. *longistaminata*.

**Table 2:** Results of analysis of enriched KEGG biological pathways of up-regulated genes in shoots and rhizomes of *Oryza longistaminata* under 7-day chilling stress.

Gene set	KEGG Term	Sample number	Background number	*P*-Value^#^
common	Plant-pathogen interaction	7	68	4.63E-10
common	Plant hormone signal transduction	4	131	0.0005
common	Spliceosome	3	106	0.0034
shoot-specific	Photosynthesis	2	78	0.033
rhizome-specific	Isoquinoline alkaloid biosynthesis	3	18	0.0007
rhizome-specific	Metabolism	4	437	0.0039
rhizome-specific	Tyrosine metabolism	3	35	0.005
rhizome-specific	Apoptosis signaling pathway	2	15	0.0106
rhizome-specific	Endocytosis	3	56	0.0182
rhizome-specific	alpha-Linolenic acid metabolism	2	28	0.0316
rhizome-specific	Cysteine and methionine metabolism	3	74	0.0376
rhizome-specific	Plant hormone signal transduction	4	131	0.0418

# Statistical method: hypergeometric test; false discovery rate correction method: Benjamini and Hochberg (1995)

### Functional categorization of genes commonly induced in shoots and rhizomes under long-term chilling stress

A total of 270 genes were identified as significantly up-regulated in both shoots and rhizomes under chilling stress (S2 Table). Of them, 33 transcription factor (TF) genes, including seven *AP2/EREBPs*, six *NACs*, four *WRKYs*, four *MYBs*, four *C2H2s*, three *GRASs*, and three *Tifys*, were highly induced (S2 Table). WRKYs are important TFs that are widely involved in the transcriptional regulation of biotic and abiotic stress responses. Four WRKY TFs, namely, *OsWRKY24 (Os01g61080)*, *OsWRKY26 (Os01g51690)*, *OsWRKY45 (Os05g25770)*, and *OsWRKY53 (Os05g27730)*, were up-regulated by chilling stress.*OsWRKY24* and *OsWRKY53* have been reported to be induced in Nipponbare under low temperature conditions [41]. In addition, *OsWRKY24*, previously identified as a new transcriptional suppressor, is able to negatively regulate GA and ABA signaling pathways in rice [42], while *OsWRKY53* functions as an elicitor in rice blast resistance to induce defensive signaling pathways in rice plants [43]. Two *OsWRKY45* alleles, *OsWRKY45-1* and *WRKY45-2*, have been found to positively regulate rice response to chilling and water-deficit stress, but have negative and positive roles, respectively, in the ABA signaling pathway in response to salt stress [44]. All these data suggest that these four chilling-induced WRKY TFs participate in the transcriptional regulation of chilling stress response in *O*. *longistaminata*.

Seven AP2/EREBP genes (*OsEATB*, *OsERF118*, *OsERF91*, Os04g57340, Os01g12440, Os04g34970, and Os02g52670) were significantly up-regulated in both shoots and rhizomes. The AP2/EREBP TF gene family comprises a large number of stress-responsive regulatory genes that play a crucial role in the transcriptional regulation of abiotic stress responses in plants [45,46]. *OsEATB*, previously characterized as an ethylene-responsive AP2/EREBP factor, can restrict rice internode elongation, but also promotes the branching potential of both tillers and spikelets by repressing the gibberellin biosynthetic gene *OsKSA* [47]. *OsERF118* is induced by different abiotic stresses including drought, salt, and cold [48]. The up-regulation of these seven AP2/EREBP genes in both shoots and rhizomes of *O*. *longistaminata* implies that these TFs have the same regulatory role in both tissues in response to chilling stress.

Previous studies have revealed that MYB and NAC TF genes are widely involved in abiotic stress tolerance [49,50,51]. According to our data, the expressions of four MYB genes (*OsMYB4*, *OsMYB30*, *OsMYB55*, and *Os01g19330*) were significantly up-regulated after 7 days of chilling stress in both studied tissues. *OsMYB4* plays an important role in the regulation of chilling stress tolerance and panicle development [52], while *OsMYB30* and *OsMYB55* are crucial regulators of plant responses to extreme temperatures and drought stress [53,54,55]. We also detected six NAC TF genes that were highly induced by chilling stress in both shoots and rhizomes of *O*. *longistaminata* plants: *OsNAC3 (Os07g12340)*, *OsNAC4 (Os01g60020)*, *OsNAC8 (Os01g15640)*, *OsNAC10 (Os11g03300)*, *OsNAC131 (Os12g03040)*, and *Os01g15640*. *OsNAC3* has been found to be positively involved in the maintenance of high water content in rice seedlings under short-term (3-h) chilling treatment [56], while *OsNAC4* and *OsNAC131* are key positive regulators of programmed cell death in rice plants under environmental stresses, especially pathogen attack [57,58]. Finally, the root-specific enhancement of *OsNAC10* expression has been found to markedly improve drought stress tolerance and to increase grain yields of rice plants [59].

We additionally identified a set of genes related to stress signaling transduction that were commonly induced in both shoots and rhizomes under long-term chilling stress; these genes included five genes encoding calmodulin-like protein (CML) (*OsCML3*, *OsCML14*, *OsCML15*, *OsCML16*, and *OsMSR2/OsCML31*), five genes encoding calcium and calmodulin-related proteins (*Os02g56310*, *Os10g28240*, *Os08g27170*, *Os12g04360*, and *Os11g04560*), and three genes encoding GIBBERELLIN-INSENSITIVE DWARF 1 (GID1) (*Os03g15270*, *Os03g57640*, and *Os01g06220*). The proteins encoded by the five CML genes are mainly composed of EF-hand Ca^2+^-binding motifs that have important and diverse roles in Ca^2+^ signaling involving regulation of cellular responses to stimuli [60,61]. Previous studies have revealed that *OsCML4* and *OsMSR2* can confer abiotic stress tolerance through reactive oxygen species scavenging and by inducing downstream genes in an ABA-dependent or independent manner [62,63]. GID1, a soluble receptor for gibberellin, is involved in the regulation of stomatal development and patterning via both ABA and GA signaling pathways in rice under drought and submergence stresses [64]. As shown in S2 Table, three *GID1* genes were evidently induced by chilling stress in our study, which implies that *GID1* may be positively involved in signaling transduction pathways related to CT in *O*. *longistaminata*.

### Genes exclusively up-regulated in aboveground shoots

TF genes with tissue-specific expression have been previously found to be highly involved in tissue-specific stress response [65,66]. In the present study, we identified 197 and 157 genes exclusively up- and down-regulated, respectively, in shoots under chilling stress (S3 Table). We noted several TF gene families among the 197 genes specifically induced in shoots, including eight AP2/EREBPs (*OsDREB1A/OsCBF3*, *OsDREB1B/OsCBF1*, *OsDREB1C/OsCBF2*, *OsDREB1G*, and *OsEREBP2*), five MYBs (*NIGT1*, *Os05g37060*, *Os02g46030*, *Os04g49450*, and *Os01g41900*) and three WRKYs (*OsWRKY10*, *OsWRKY64*, and *OsWRKY76*). Three of these genes, *OsDREB1A*, *OsDREB1B*, and *OsDREB1C*, have been previously characterized as transcription activators functioning in abiotic stress response [14] and have been identified as the central regulators of cold stress-responsive transcription pathways in rice [15]. Over-expression of *OsDREB1G* can evidently enhance rice tolerance to water deficit stress [67]. The *OsEREBP2* gene has been identified as a multifunctional TF putatively involved in several stress responses in rice [68]. The induced expression of these AP2/EREBP genes under long-term low-temperature conditions implies that the CBF regulon and other AP2/EREBP TF genes play a unique role in shoot tolerance to chilling stress.

MYB and WRKY are also important TFs that function in a variety of plant-specific processes including responses to biotic and abiotic stresses [69,70]. We found five MYB TFs, including *NIGT1*, that were evidently induced only in shoots. *NIGT1* (Os02g22020) is a nitrate-inducible and auto-repressible transcriptional repressor that may play a role in nitrogen response in rice [71]. *OsWRKY10* is involved in the transcriptional activation of defense-related genes in response to rice pathogens [72]. *OsWRKY62* and *OsWRKY76* function as negative regulators of biosynthetic defense-related metabolites [73] and play dual and opposing roles in blast disease resistance and cold tolerance [74].

Consistent with the results of the KEGG analysis, six genes related to photosynthesis were uniquely induced in shoots by chilling stress (S3 Table); these genes encode two photosynthetic reaction center proteins, two photosystem I assembly protein YCF4s, and two photosystem II reaction center Hs proteins. All these proteins are important components of the major multi-subunit protein complexes involved in the process of photosynthesis [75]. A large amount of evidence has revealed that photosynthesis-associated genes are highly involved in abiotic stress response, and, in most cases, are evidently down-regulated in response to environmental stresses [75]. The induced expression of photosynthesis-related genes in shoots, however, may alleviate the detrimental effect of long-term chilling stress on the photosynthetic complex in rice plants, an idea in keeping with the observation that enhancement of photosynthetic performance is associated with cold acclimation in overwintering crops [40].

### Genes uniquely differentially expressed in rhizomes under chilling stress

A total of 198 and 173 genes were respectively identified as up- or down-regulated exclusively in rhizomes under chilling stress (S4 Table). GO analysis functionally classified the 198 up-regulated genes into the categories of response to stress (*n* = 29), transcription regulation (*n* = 21), binding (*n* = 19), metabolism (*n* = 33), transport (*n* = 7), redox homeostasis (*n* = 8), and cell and cell cycle (*n* = 9), in addition to genes with unknown function.

Among the 21 genes functionally associated with transcriptional regulation, six AP2/EREBPs (*OsERF922*, *Os02g43820*, *Os07g12510*, *Os08g45110*, *Os09g39850*, and *Os06g11860*), three NACs (*OsNAC9*, *Os01g48446*, and *Os05g10620*), and two WRKYs (*OsWRKY25* and *OsWRKY74*) were evidently induced in rhizomes by chilling stress. The six AP2/EREBP genes distinctively induced by chilling in rhizomes were a different subset of AP2/EREBP genes than those described above as induced in shoots.*OsERF922* has been found to be a negative regulator of defense against the rice blast fungus *Magnaporthe oryzae* and salt stress [11,76]. *OsNAC9* is involved in root development; specifically, enhanced expression of this gene in transgenic rice roots can alter root architecture, thereby conferring improved drought tolerance and grain yield [77]. Furthermore, ectopic over-expression of *OsNAC9* (*SNAC1*) in cotton is able to improve drought and salt tolerance by enhancing root development in transgenic cotton plants [78]. The *OsWRKY74* gene has been demonstrated to enhance phosphate starvation tolerance as well as cold tolerance [79]. The fact that all these TF genes were specifically induced in rhizomes by chilling stress suggests that they play unique regulatory roles in underground organs/rhizomes against long-term low-temperature stress.

Eight genes related to redox homeostasis were evidently up-regulated in rhizomes: three encoding glutathione S-transferase GSTU6, two encoding oxidoreductase, and one each encoding OsTrx23, peroxidase, and heavy metal transport/detoxification protein. *OsGSTU* family genes have been characterized as functioning in abiotic stress responses by preventing oxidative damage by reactive oxygen species [80,81], while OsTrx23 is involved in redox regulation in plants under abiotic stress [82]. The highly induced expression of these genes implies that antioxidant capacity may be enhanced in rhizomes under chilling stress.

### Comparative analysis of the chilling-induced degs in *o*. *longistaminata* and two cultivated rice varieties

In a previous study, we comparatively analyzed transcriptome changes of two cultivated rice genotypes (chilling tolerant LTH and chilling sensitive IR29) under time-series (2-, 8-, 24- and 48-h) chilling stress [18]. The DEGs in both genotypes under 48-h chilling stress were comparatively analyzed in the present study. A combined set of 1,069 DEGs (665 up-regulated and 403 down-regulated) in shoots and rhizomes of *O*. *longistaminata* were compared with chilling-induced DEGs in LTH and IR29. As shown in S2 Fig, a substantial number of genes were found to be genotype-specifically regulated by chilling stress. Strikingly, the majority of up-regulated (447 out of 665) and down-regulated (295 out of 403) genes in *O*. *longistaminata* were identified as genotype-exclusive. Further KEGG analysis of the up-regulated genes in the three genotypes revealed that genes involved in several biological pathways, including spliceosome, apoptosis signaling and tyrosine metabolism pathways, were highly enriched in *O*. *longistaminata* (S5 Table). This result indicates their unique role in *O*. *longistaminata* chilling stress response. Previous studies have shown that cultivated and wild rice exhibit considerable intraspecific variation in chilling tolerance [7,8], and diverse genotypes with contrasting CTs have distinct transcriptome alterations in response to chilling stress [18]. In the present study, many DEGs in *O*. *longistaminata* were characterized as being exclusively regulated by chilling stress, implying their critical role in chilling stress tolerance of *O*. *longistaminata*.

### *Cis*-regulatory element (cre) analysis of degs in shoots and rhizomes under chilling stress

CREs play important roles in spatial and temporal transcriptional regulation of genes in response to environmental stresses [83]. In this study, we investigated the CREs of chilling-induced genes specifically identified in *O*. *longistaminata* shoots and rhizomes. Several CREs, such as AAAG, (CT)ACT, CA(ACGT)(ACGT)TG, (ACGT)GATT, ACGT, TGAC, and GATA, showed evidently higher abundance in shoots and rhizomes (Table 3). Of these CREs, the AAAG motif is a binding site of the Dof TF, which determines guard cell-specific expression [84], while the (CT)ACT motif is a CRE for mesophyll-specific gene expression [85]. The CRE of CA(ACGT)(ACGT)TG, identified as an ICE1-binding site, is involved in chilling stress response [86]. The GATT motif is the specific binding element of type-B response regulators, which are widely involved in the cytokinin signal transduction pathway [87]. The ACGT motif is involved in dehydration stress-triggered up-regulation and dark-induced senescence [88]. The TGAC motif, a core sequence of the W-box, is essential to WRKY function and is highly involved in pathogen defense response [89]. The GATA element is necessary for phytochrome regulation [90]. All these CREs were highly enriched in promoter regions of up-regulated genes in shoots and rhizomes, thus implicating multiple regulatory genes and complex genetic networks in the transcriptional regulation of *O*. *longistaminata* CT.

**Table 3:** *Cis*-regulatory elements identified in induced genes in shoots and rhizomes under 7-day chilling stress.

*Cis*-element	DEGs	Shoot Specific	Rhizome Specific	Function
	No. of tested genes	137	153	
AAAG	Total (%)	97.1	99.3	Dof gene binding
	Two or more copies (%)	91.2	94.8	
(CT)ACT	Total (%)	100	99.3	mesophyll-specific gene expression
	Two or more copies (%)	100	96.7	
CA(ACGT)(ACGT)TG	Total (%)	97.8	98.7	cold response; ICE1 binding, CBF/DREB1
	Two or more copies (%)	90.5	89.5	
(ACGT)GATT	Total (%)	97.1	96.1	ARR1, Type-B response regulators binding
	Two or more copies (%)	92.0	86.9	
ACGT	Total (%)	83.2	88.2	induction by dehydration stress
	Two or more copies (%)	61.3	69.9	
TGAC	Total (%)	91.2	94.8	W box, WRKY binding, defense response
	Two or more copies (%)	75.9	78.4	
GATA	Total (%)	92.0	92.8	chlorophyll a/b binding protein
	Two or more copies (%)	79.6	79.1	

### AS of transcripts in shoots and rhizomes under chilling stress

AS plays a crucial role in plant development and response to environmental stimuli including biotic and abiotic stresses [91,92]. To explore genome-wide AS in shoots and rhizomes under 7-day chilling stress conditions, we assembled and comparatively analyzed all transcripts. As shown in Table 4, 14,523 genes associated with 76,000 AS events were detected in shoots and rhizomes under chilling stress and control conditions. A total of 9,717/9,351 and 8,627/8,691 genes were identified as alternatively spliced in shoots/rhizomes under chilling stress and control conditions, respectively, which indicates that the number of genes undergoing AS was higher in shoots and rhizomes under chilling stress. A total of 28,678/28,411 and 22,871/23,155 AS events were accordingly identified in shoots/rhizomes under chilling stress and control conditions, respectively. All these results demonstrate that the frequency of AS events was greatly increased by chilling stress treatment in both shoots and rhizomes.

**Table 4:** Alternative splicing statistics for shoots and rhizomes of *Oryza longistaminata* under chilling stress and control growth conditions.

Classification of alternative splicing events	Shoots under chilling stress	Shoots under control condition	Rhizomes under chilling stress	Rhizomes under control condition	Total
Alternative Acceptor Site (AAS)	8984	7196	8583	7153	22957
Alternative Donor Site (ADS)	6747	6158	6831	6155	17906
Exon Skip (ES)	1944	2243	2013	2216	6207
Exon New (EN)	5543	4060	5430	4086	14634
Intron Retained (IR)	5460	3214	5554	3545	14296
Total alternative splicing events/genes	28678/9717	22871/8627	28411/9351	23155/8691	76000/14532

A previous study found that plants can produce diverse transcripts with different functions by AS in response to environmental stresses [92]. To investigate the relationship between gene expression alteration and AS, we comparatively analyzed DEG and AS data in shoots and rhizomes under chilling stress. As shown in S6 Table, 191/189 up-regulated and 13/15 down-regulated genes were alternatively spliced in shoots/rhizomes under chilling stress, which indicates that the majority of AS events occurred in the up-regulated genes under stress. Of them, 80 up-regulated genes and 1 down-regulated one were common to both shoots and rhizomes (S7 Table, Fig 3). GO analysis revealed that these 80 genes were functionally enriched in the transcription regulation category, which suggest that AS is involved in positive regulation of transcription networks in both shoots and rhizomes under long-term chilling stress.

**Fig 4:**
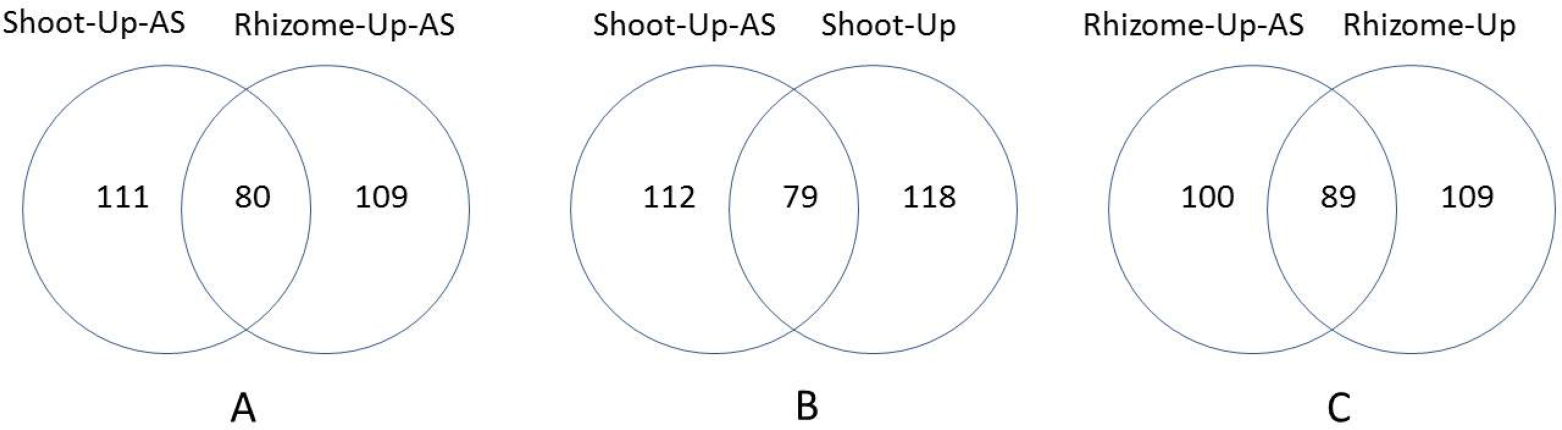
Venn diagram of organ-specific up-regulated genes with alternative splicing (AS) events in *Oryza longistaminata* under 7-day chilling stress conditions. Shoot-Up-AS, Rhizome-Up-AS, Shoot-Up, and Rhizome-Up refer to up-regulated genes with AS in shoots, up-regulated genes with AS in rhizomes, specifically up-regulated genes in shoots, and specifically up-regulated genes in rhizomes, respectively, under chilling stress.

We further analyzed organ-specific DEGs with AS events. As shown in Fig 4, 79 shoot-specific and 89 rhizome-specific up-regulated genes were found to be alternatively spliced under chilling stress. GO analysis found that the 79 shoot-specific up-regulated genes with AS were functionally enriched in the categories of regulation of gene expression and photosynthesis. Several TF genes were noted, including *OsWRKY64*, *OsWRKY76*, *OsDREB1G*, *OsHsfA9*, *OsEREBP2*, and a gene encoding AP2 domain containing protein (Os08g36920) and a gene encoding homeobox associated leucine zipper (Os06g48290); this indicates that AS may play a unique role in the regulation of TF genes in shoots in response to chilling stress. We also detected 89 rhizome-specific up-regulated genes that were functionally enriched in calcium ion binding. Several genes encoding EF hand family proteins (Os11g38780, Os03g19720, and Os07g12240) and phospholipase D (Os03g02740) were noted. All these data demonstrate that AS is involved in regulating CT in an organ-specific manner.

Previous studies have revealed that AS is highly involved in the post-transcriptional regulation of cold acclimation and CT in plants. Circadian clock associated 1 (CCA1) and late elongated hypocotyl (LHY), the key components of the circadian clock in Arabidopsis, are related to the transcriptional regulation of genes of the CBF cold-response pathway [93]. In one study, over-expression of the alternative transcripts of *CCA1*, *CCA1α*, and *CCA1β* was found to enhance and reduce cold tolerance of the corresponding transgenic plants [94], indicating that cold-induced AS contributed to low temperature stress tolerance. In our study, seven TF genes, namely, four *AP2/EREBPs*, two *WRKYs*, and *OsHsfA9*, were evidently induced and coincidently alternatively spliced in shoots under chilling stress. Highly accumulated transcript variants of these TF genes may have diverse functions in regulating downstream genes of genetic networks of chilling stress tolerance in shoots. Finally, three genes encoding calcium ion binding proteins and the phospholipase gene are related to stress signaling transduction [95,96]. Enhanced AS variants of these genes may specifically affect the calcium-mediated signaling cascade in rhizomes in response to chilling stress.

## Conclusions

Our physiological analysis revealed that an increase in osmoprotectants and antioxidants in shoots and rhizomes under chilling stress could enhance the CT of *O*. *longistaminata* by stabilizing biological components and maintaining redox homeostasis. Numerous genes were identified as exclusively differentially expressed in shoots and rhizomes of *O*. *longistaminata* under 7 days of chilling treatment. These genes were functionally involved in diverse molecular pathways closely related to transcriptional and post-transcriptional regulatory cascades and environmental adaptation, thus implying that the genetic mechanism of CT in *O*. *longistaminata* is complex. The present study represents the first comprehensive survey of tissue-specific transcriptome alterations of *O*. *longistaminata* in response to long-term chilling stress using RNA sequencing. The chilling-responsive genes identified in this study are putative candidates for further functional confirmation and have potential application to molecular breeding-based improvement of CT in crops.

## Supporting information

S1 Table Information on primers used in qRT-PCR analyses. (XLSX)

S2 Table Genes induced in both shoots and rhizomes of *Oryza longistaminata* under 7-day chilling stress. (XLSX)

S3 Table Genes specifically differentially regulated in shoots under 7-day chilling stress. (XLSX)

S4 Table Genes specifically differentially regulated in rhizomes of *Oryza longistaminata* under 7-day chilling stress. (XLSX)

S5 Table Analysis of enriched KEGG biological pathways of up-regulated genes in *Oryza longistaminata* and cultivated rice varieties LTH and IR29 under chilling stress. (XLSX)

S6 Table Shoot- and rhizome-specific differentially expressed genes with alternative splicing events. (XLSX)

S7 Table Genes with alternative splicing induced in both shoots and rhizomes under chilling stress. (XLSX)

S1 Fig Validation of RNA sequencing (RNA-seq) results using quantitatve real-time PCR (RT-PCR) assays. The genes were randomly selected from differentially expressed genes in shoots and rhizomes of *Oryza longistaminata* under 7-d chilling stress. Information of primers is provided in S1 Table. Sample numbers 1–4 indicate material collected from shoots under control condition, shoot sunder chilling stress, rhizomes under control condition and rhizomes under chilling stress, respectively. Left and right y-axes indicate relative expression levels detected by qRT-PCR and RNA-seq, respectively. Transcript expression levels were normalized against endogenous *Actin* transcripts.
(PPTX)

S2 Fig Venn diagram analysis of chilling-induced differentially expressed genes (DEGs) in *Oryza longistaminata* and cultivated rice varieties Lijiangxintuanhegu (LTH) and IR29. Data for DEGs of LTH and IR29 under chilling stress are from our previous study (Zhang et al., 2012. Comparative transcriptome profiling of chilling stress responsiveness in two contrasting rice genotypes. PLoS ONE. 7(8):e43274.)
(PPTX)
