# Supplementary material for: Differential transcriptome profiling of chilling stress response between shoots and rhizomes of Oryza longistaminata using RNA sequencing
Source: PLoS One. 2017 Nov 30;12(11):e0188625. doi: 10.1371/journal.pone.0188625 (PMC5708648; doi:10.1371/journal.pone.0188625)
Supplement: S1 Fig — The genes were randomly selected from differentially expressed genes in shoots and rhizomes of Oryza longistaminata under 7-d chilling stress. Information of primers is provided in S1 Table. Sample numbers 1–4 indicate material collected from shoots under control condition, shoot sunder chilling stress, rhizomes under control condition and rhizomes under chilling stress, respectively. Left and right y-axes indicate relative expression levels detected by qRT-PCR and RNA-seq, respectively. Transcript expression levels were normalized against endogenous Actin transcripts. (PPTX) [file pone.0188625.s008.pptx]

## Slide 1
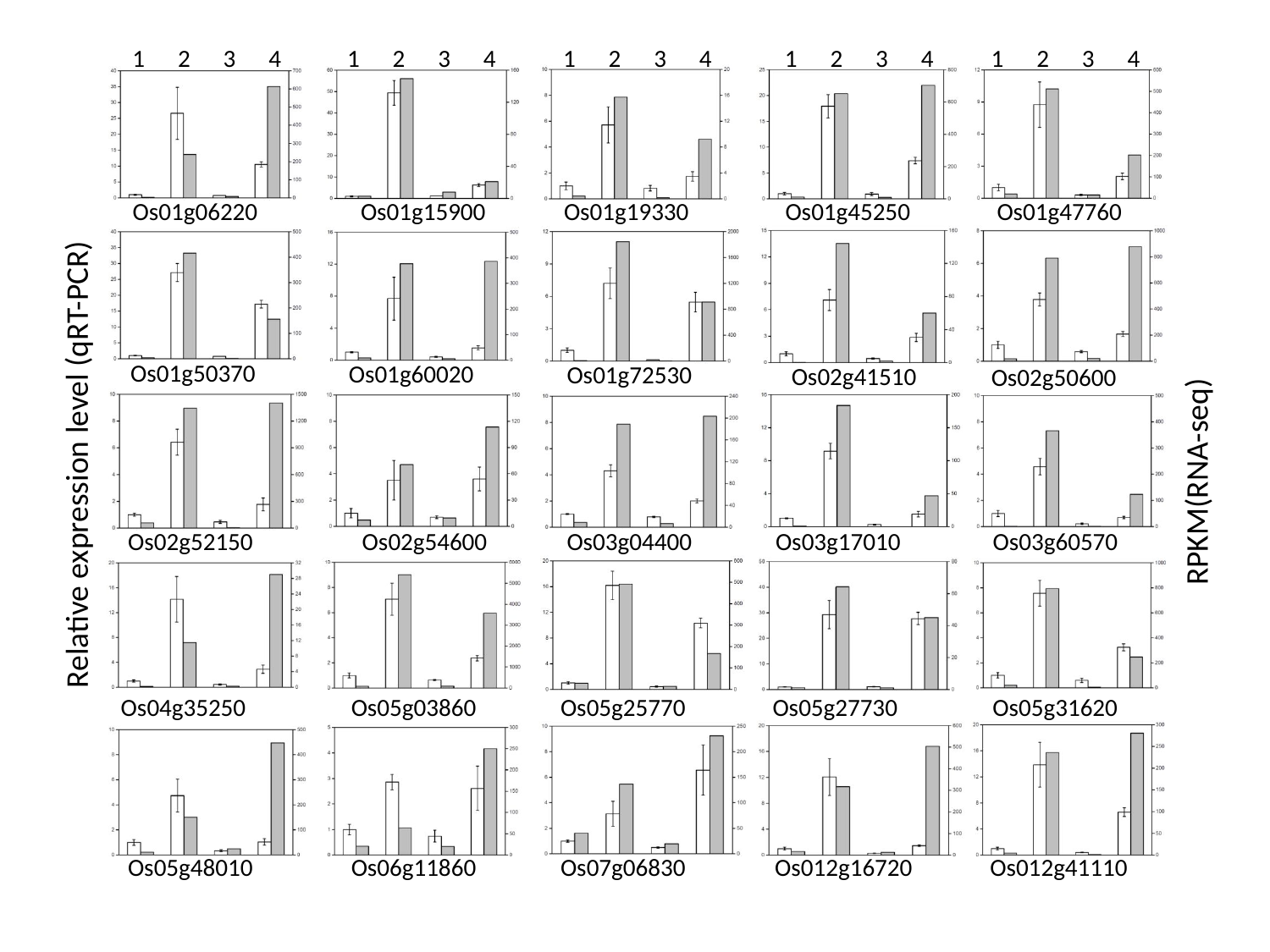

1 2 3 4
1 2 3 4
1 2 3 4
1 2 3 4
1 2 3 4
Os01g06220
Os01g15900
Os01g19330
Os01g45250
Os01g47760
RPKM(RNA-seq)
Os01g50370
Os01g60020
Os01g72530
Os02g41510
Os02g50600
Relative expression level (qRT-PCR)
Os02g52150
Os02g54600
Os03g04400
Os03g17010
Os03g60570
Os04g35250
Os05g03860
Os05g25770
Os05g27730
Os05g31620
Os05g48010
Os06g11860
Os07g06830
Os012g16720
Os012g41110

## Slide 2
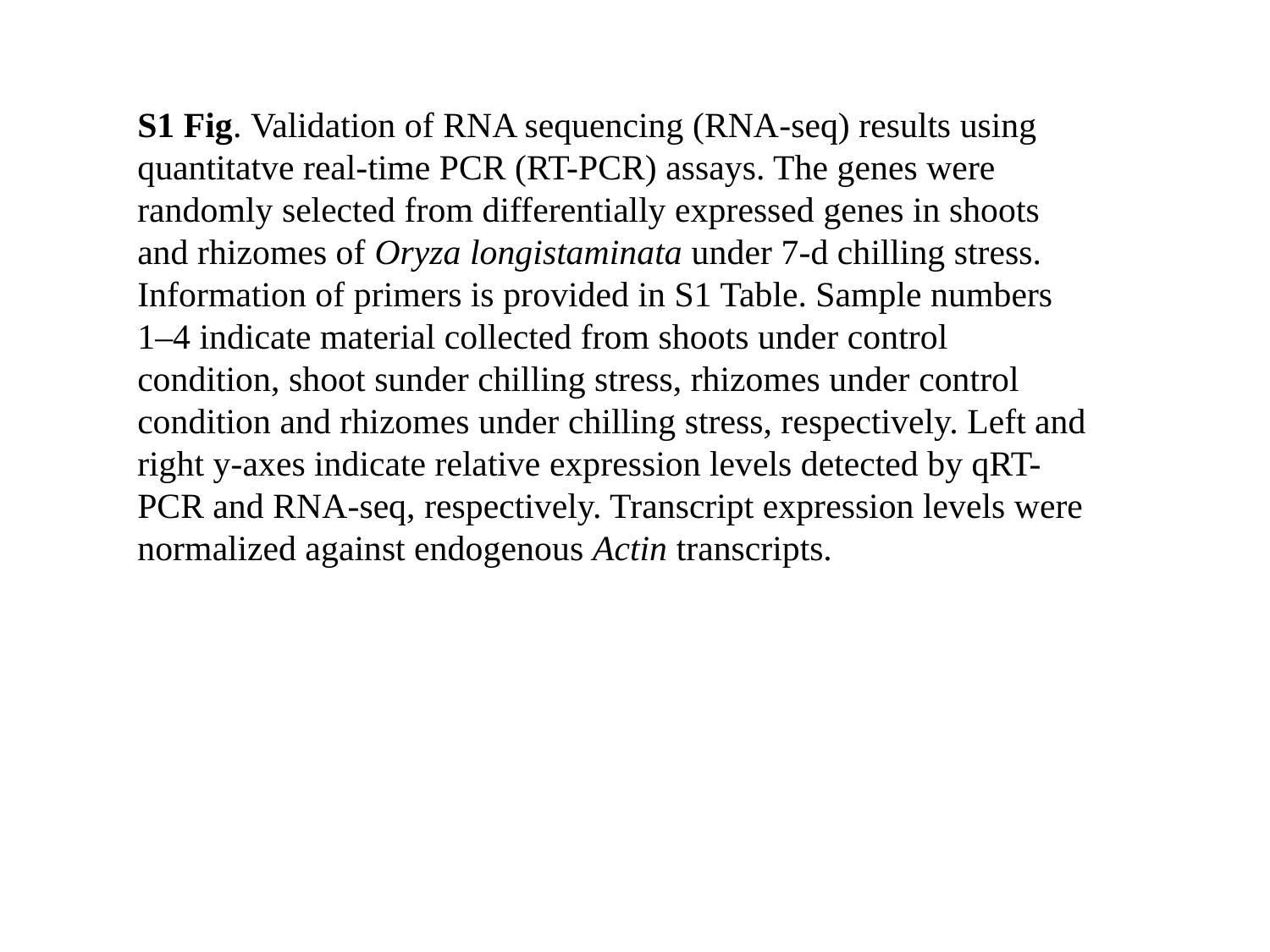

S1 Fig. Validation of RNA sequencing (RNA-seq) results using quantitatve real-time PCR (RT-PCR) assays. The genes were randomly selected from differentially expressed genes in shoots and rhizomes of Oryza longistaminata under 7-d chilling stress. Information of primers is provided in S1 Table. Sample numbers 1–4 indicate material collected from shoots under control condition, shoot sunder chilling stress, rhizomes under control condition and rhizomes under chilling stress, respectively. Left and right y-axes indicate relative expression levels detected by qRT-PCR and RNA-seq, respectively. Transcript expression levels were normalized against endogenous Actin transcripts.
