# Supplementary material for: Differential transcriptome profiling of chilling stress response between shoots and rhizomes of Oryza longistaminata using RNA sequencing
Source: PLoS One. 2017 Nov 30;12(11):e0188625. doi: 10.1371/journal.pone.0188625 (PMC5708648; doi:10.1371/journal.pone.0188625)
Supplement: S2 Fig — Data for DEGs of LTH and IR29 under chilling stress are from our previous study (Zhang et al., 2012. Comparative transcriptome profiling of chilling stress responsiveness in two contrasting rice genotypes. PLoS ONE. 7(8):e43274.) (PPTX) [file pone.0188625.s009.pptx]

## Slide 1
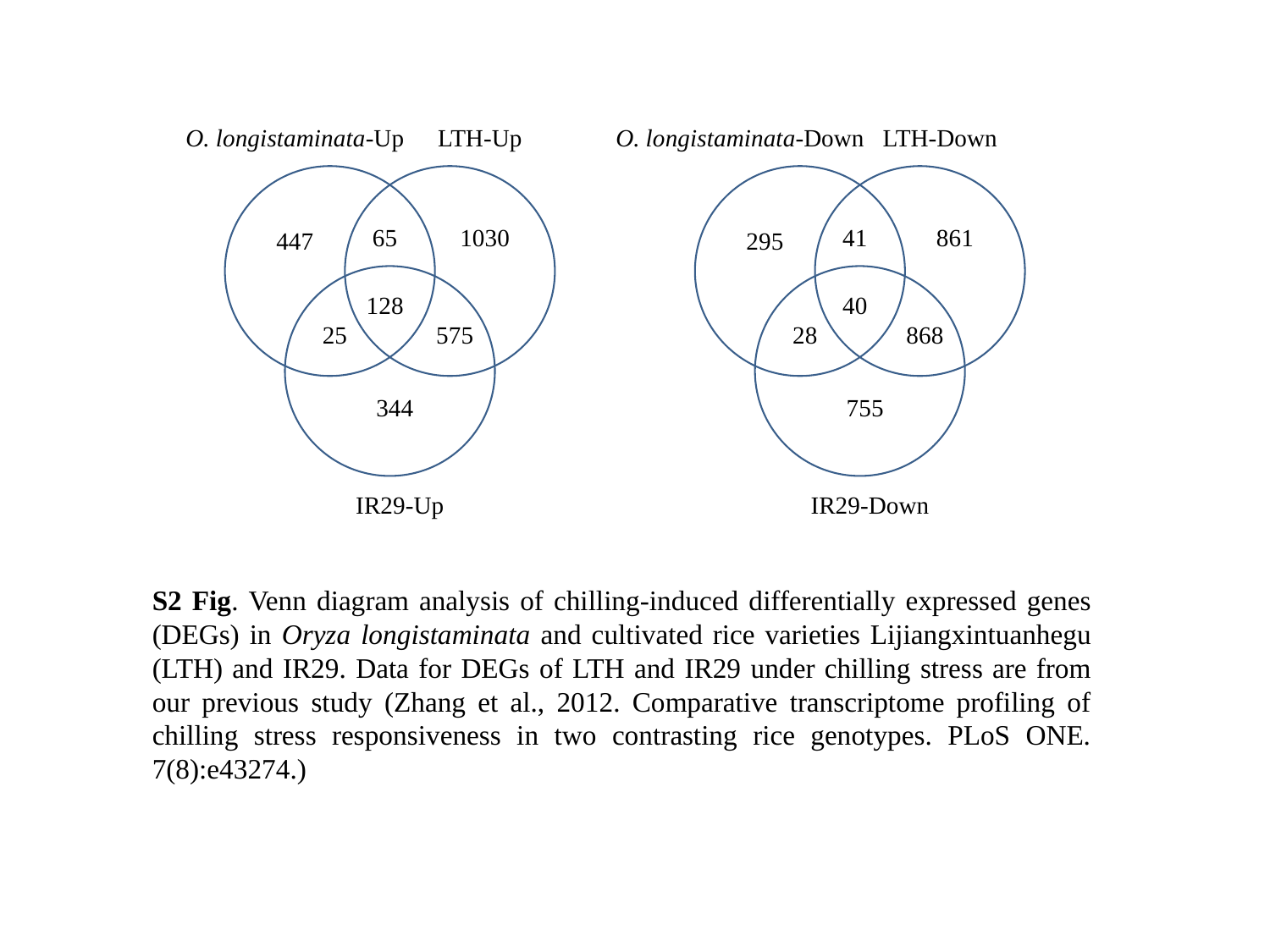

O. longistaminata-Up
LTH-Up
O. longistaminata-Down
LTH-Down
65
1030
41
861
447
295
128
40
25
575
28
868
344
755
IR29-Up
IR29-Down
S2 Fig. Venn diagram analysis of chilling-induced differentially expressed genes (DEGs) in Oryza longistaminata and cultivated rice varieties Lijiangxintuanhegu (LTH) and IR29. Data for DEGs of LTH and IR29 under chilling stress are from our previous study (Zhang et al., 2012. Comparative transcriptome profiling of chilling stress responsiveness in two contrasting rice genotypes. PLoS ONE. 7(8):e43274.)
